# Reproductive and genetic roles of the maternal progenitor in the origin of common wheat (*Triticum aestivum* L.)

**DOI:** 10.1002/ece3.6985

**Published:** 2020-12-02

**Authors:** Yoshihiro Matsuoka, Naoki Mori

**Affiliations:** ^1^ Fukui Prefectural University Yoshida Fukui Japan; ^2^ Crop Evolution Graduate School of Agricultural Science Kobe University Kobe Japan

**Keywords:** *Aegilops tauschii*, allopolyploidy, hybrid genome doubling, interspecific hybridization, nonreductional meiosis, *Triticum*, unreduced gametes

## Abstract

Common wheat (*Triticum aestivum* L., AABBDD genome) is thought to have emerged through natural hybridization between *Triticum turgidum* L. (AABB genome) and *Aegilops tauschii* Coss. (DD genome). Hybridization barriers and doubling of the trihaploid F_1_ hybrids’ genome (ABD) via unreduced gamete fusion had key roles in the process. However, how *T. turgidum*, the maternal progenitor, was involved in these mechanisms remains unknown. An artificial cross‐experiment using 46 cultivated and 31 wild *T. turgidum* accessions and a single *Ae. tauschii* tester with a very short genetic distance to the common wheat D genome was conducted. Cytological and quantitative trait locus analyses of F_1_ hybrid genome doubling were performed. The crossability and ability to cause hybrid inviability did not greatly differ between the cultivars and wild accessions. The ability to cause hybrid genome doubling was higher in the cultivars. Three novel *T. turgidum* loci for hybrid genome doubling, which influenced unreduced gamete production in F_1_ hybrids, were identified. Cultivated *T. turgidum* might have increased the probability of the emergence of common wheat through its enhanced ability to cause genome doubling in F_1_ hybrids with *Ae. tauschii*. The ability enhancement might have involved alterations at a relatively small number of loci.

## Graphical abstract

Common wheat (*Triticum aestivum* L., AABBDD genome) is thought to have emerged through natural hybridization between *Triticum turgidum* L. (AABB genome) and *Aegilops tauschii* Coss. (DD genome). Doubling of the trihaploid F_1_ hybrids’ genome (ABD) via unreduced gamete fusion had a key role in the process. We report on three novel *T. turgidum* loci for hybrid genome doubling that might have increased the probability of the emergence of common wheat.

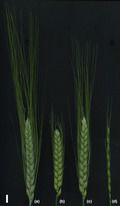

## 1 INTRODUCTION

Common wheat (*Triticum aestivum* L.; AABBDD genome) is the most widely grown crop worldwide. Its origin and evolution have been matters of long‐standing research interest. Currently, common wheat is thought to have emerged through natural hybridization of *Triticum turgidum* L. (AABB genome) as the maternal progenitor and *Aegilops tauschii* Coss. (formerly known as *Aegilops squarrosa* L.; DD genome) as the paternal progenitor (Kihara, 1944; McFadden & Sears, 1944). *T. turgidum* exists in its wild form [*T. turgidum* subsp. *dicoccoides* (Körn. ex Asch. & Graebn.) Thell.] and as various cultivars, whereas *Ae. tauschii* is a wild species that is spread throughout central Eurasia, from Syria to western China (Kilian et al., 2011; van Slageren, 1994). The domestication of *T. turgidum* took place in the Fertile Crescent ca. 10,000 years ago (Zohary et al., 2012). In a widely accepted scenario for the origin of common wheat, cultivated *T. turgidum*, which met with *Ae. tauschii* through human‐mediated migration associated with the spread of agriculture across and beyond the Fertile Crescent, is considered the female progenitor, based mainly on the fact that no wild form of *T. aestivum* has ever been found (Kihara, 1966, 1975). The *T. turgidum* cultivars are classified into two types based on grain threshability: the hulled type, including emmer wheat [*T. turgidum* subsp. *dicoccum* (Schrank ex Schübl.) Thell.] and the free‐threshing type, including durum wheat [*T. turgidum* subsp. *durum* (Desf.) Huns.]. Emmer wheat is prototypic. Lines of genetic and archeological evidence suggest that the female progenitor of common wheat likely was a free‐threshing cultivar (Matsuoka, 2011). The location in which the original hybridization took place is unknown; however, the southern coastal region of the Caspian Sea is a good candidate based on genetic evidence (Matsuoka & Takumi, 2017; Tsunewaki, 1966; Wang et al., 2013).

The evolution of common wheat represents a typical case of plant allopolyploid evolution in which hybridization barriers between the parental species and unreduced gamete production in their F_1_ hybrids are highly influential (Ramsey & Schemske, 1998). Pre‐ and postpollination mechanisms can prevent allopolyploid speciation from occurring by limiting the formation and viability of interspecific F_1_ hybrids, whereas the fusion of unreduced gametes results in the formation of nascent allopolyploids through doubling of the F_1_ hybrid genome. In crosses of *T. turgidum* with *Ae. tauschii*, various hybridization barriers, such as reduced crossability between the parental species and lethality/weakness in the F_1_ hybrids, are observed (Matsuoka & Takumi, 2017; Mizuno et al., 2010, 2011; Nishikawa, 1953). The trihaploid F_1_ hybrids (ABD genome) undergo genome doubling through unreduced gamete fusion and set allohexaploid F_2_ seeds (AABBDD; Kihara, 1946). The genotypes of parental *T. turgidum* and *Ae. tauschii* accessions influence the frequency of genome doubling, which can be measured as the seedset rates of F_1_ hybrids (Fukuda & Sakamoto, 1992; Kihara et al., 1965).

Previous studies have shed some light on the role of *Ae. tauschii* in the evolution of common wheat. *Ae. tauschii* has three intraspecific lineages that are defined on the basis of genome‐wide molecular marker genotypes: two major lineages (TauL1 and TauL2) and one small lineage (TauL3). TauL2 and TauL3 are more closely related to the D genome of common wheat than TauL1 (Matsuoka et al., 2013; Mizuno et al., 2010). Geographically, TauL1 is spread across the species range, whereas TauL2 is restricted to the western region of the species range, that is, the Transcaucasian‐Middle Eastern region, and TauL3 exists only in Georgia. Interestingly, *Ae. tauschii* is polymorphic in its ability to cause reproductive barrier expression in the cross with *T. turgidum* and unreduced gamete production in F_1_ hybrids (Matsuoka et al., 2007). This suggests that some genotypes of *Ae. tauschii* may have had more opportunities to be involved in the origin of common wheat than others because they frequently hybridized with *T. turgidum* and because the F_1_ hybrids were likely to produce unreduced gametes. In fact, artificial cross‐studies have shown that southern Caspian accessions of the TauL2 lineage have a high potential for natural hybridization with *T. turgidum* (Matsuoka & Takumi, 2017). Thus, the genealogically and geographically structured polymorphism in the crossability with *T. turgidum* may have had a profound impact on the spatial patterns of common wheat speciation. In addition, *Ae. tauschii* has six quantitative trait loci (QTLs) for genome doubling in the F_1_ hybrids it produces with *T. turgidum* (Matsuoka et al., 2013).

However, very little is known about the natural variation in *T. turgidum's* ability to cause reproductive barrier expression in crosses with *Ae. tauschii*. In addition, how variation in the ability to cause genome doubling in F_1_ hybrids with *Ae. tauschii* is structured in this species remains to be addressed. Previous artificial cross‐studies have shown that the *T. turgidum* genotype influences the degrees of viability and fertility in F_1_ hybrids with *Ae. tauschii* (Kihara et al., 1965; Nishikawa, 1953). *T. turgidum* has a QTL that is involved in unreduced gamete production in the hybrids (Hao et al., 2014). Nevertheless, further studies are required to clarify the role of *T. turgidum* in the evolution of common wheat and to answer other questions such as: Do cultivated and wild forms differ in reproductive isolation patterns in crosses with *Ae. tauschii*? Do they differ in the ability to cause hybrid inviability and genome doubling? What is the genetic basis of the ability to cause hybrid genome doubling in this species? Answers to these questions are essential to understand the genetic underpinning of common wheat evolution.

In the present study, a series of artificial cross‐experiments, QTL analysis, and cytological observations were conducted to address the above questions. In the artificial cross‐experiment, a diverse array of *T. turgidum* accessions were crossed with a single *Ae. tauschii* tester accession with a very short genetic distance to the D genome of common wheat, with the aim to evaluate the natural variation patterns of crossability with *Ae. tauschii* and the abilities to cause inviability and genome doubling in F_1_ hybrids. The QTL analysis and cytological observations were conducted to clarify the genetic basis of the ability of *T. turgidum* to cause genome doubling in F_1_ hybrids with *Ae. tauschii*. Major findings were as follows: (a) the cultivars were less crossable with the tester than with the wild accessions were, but the crossability value ranges widely overlapped between the two groups, (b) the proportion of the accessions that produced viable, fertile F_1_ plants was similar in cultivars and wild accessions, (c) hybrid genome doubling frequencies were generally increased in cultivar‐derived F_1_ versus wild‐accession‐derived F_1_ genotypes, (d) some cultivar‐derived F_1_ genotypes had very high hybrid genome doubling frequencies (>50%), and (e) in the cultivars, a relatively small number of loci that influence unreduced gamete production in F_1_ hybrids with *Ae. tauschii* might be involved in the enhanced ability to cause hybrid genome doubling. On the basis of these findings, we discuss insights into the role of *T. turgidum* in the evolution of common wheat.

## 2 MATERIALS AND METHODS

### 2.1 *T. turgidum* accessions

This study used 46 cultivated and 31 wild *T. turgidum* accessions (Table S1). The cultivated accessions represented a diverse array of intraspecific taxa: *T. turgidum* subsp. *carthlicum* (Nevski) Á. Löve & D. Löve (one accession), *T. turgidum* subsp. *dicoccum* (Schrank ex Schübl.) Thell. (10 accessions), *T. turgidum* subsp. *durum* (Desf.) Husn. (24 accessions), *T. turgidum* subsp. *paleocolchicum* (Menabde) Á. Löve & D. Löve (one accession), *T. turgidum* subsp. *turanicum* (Jakubz.) Á. Löve & D. Löve (one accession), and *T. turgidum* subsp. *turgidum* L. (two accessions). In addition, seven cultivated accessions that were classified by their collectors as *Triticum abyssinicum* Vav. (two accessions), *Triticum ispahanicum* Heslot (two accessions), *Triticum orientale* Perc. (one accession), and *Triticum pyramidale* (Del.) Perc. (two accessions) were used. All the cultivated accessions were landraces, except for the modern cultivar, *T. turgidum* subsp. *durum* cv. “Langdon” (called LDN here after). Wild accessions (*T. turgidum* subsp. *dicoccoides*) were sampled to represent the subspecies distribution range and chloroplast DNA haplotype diversity. Thirty of the 32 chloroplast DNA haplotypes found in a source collection were included in the present study (Mori et al., 2003).

### 2.2 *Ae. tauschii* tester accession

The use of an appropriate *Ae. tauschii* tester is essential for the artificial cross‐experiment to be informative with regard to the role of *T. turgidum* in the evolution of common wheat. In the present study, we used a single *Ae. tauschii* accession, KU‐2103, as the tester. This accession belongs to the TauL2 lineage (Matsuoka et al., 2013), and it was provided by the Plant Germ‐plasm Institute of Kyoto University and the National BioResource Project (NBRP)–KOMUGI. We used KU‐2103 as the tester because (a) it is one of the accessions that had the shortest genetic distance to the D genome of common wheat (Wang et al., 2013), (b) it has positive alleles at the major QTLs that are involved in the expression of genome doubling in F_1_ hybrids with LDN (Matsuoka et al., 2013), and (c) it originated in the southern coastal Caspian region of Iran, which is a candidate region for the cradle of common wheat (Kihara et al., 1965, reported as “stock number 2148” therein). KU‐2013 was also used to produce a segregant population for the QTL analysis.

### 2.3 Artificial cross‐experiment

Seeds of the *T. turgidum* and *Ae. tauschii* accessions were sown in individual pots in early winter, and the germinated plants grew in an unheated greenhouse throughout the season. Spikes of healthy *T. turgidum* plants were fully emasculated before anthesis and individually bagged until use, to prevent pollen contamination. Two days after emasculation, pollen of the KU‐2013 tester was applied to the pistils of the emasculated spikes by hand. Only pistils on the first and second florets of well‐developed spikelets were pollinated. Pollen of one dehiscing anther was applied to up to two pistils. Multiple spikes were used for crossing in all *T. turgidum* accessions, except for KU‐145, PI 254190, and Vernal. Spikes were immediately rebagged after pollination. Gibberellic acid solution was not applied to the embryos. To reduce the effect of possible technical idiosyncrasies, a single person performed emasculation and pollination. After harvest, the well‐developed seeds per spike were counted. The crossability value was calculated for each *T. turgidum* accession as the number of seeds set divided by the number of pollinated florets.

The F_1_ hybrid seeds were germinated in Petri dishes at 20°C, transplanted into individual pots in early winter, and grown in an unheated greenhouse. Up to six seeds were used per F_1_ genotype. Colchicine was not applied to the F_1_ plants. Hybridity was confirmed morphologically by comparing coleoptile color, waxiness, and spike shape between the F_1_ and their maternal parent plants. For selfing, spikes on well‐developed tillers were individually bagged before anthesis. After harvest, well‐developed seeds set in the first and second florets of each spike were counted. For each F_1_ genotype, the hybrid genome doubling frequency was determined as the selfed seedset rate, as seedset by selfing is a good indicator of the occurrence of genome doubling via unreduced gamete fusion (Kihara, 1946). The hybrid genome doubling frequency was calculated for each F_1_ genotype as the number of seeds set divided by the number of florets examined for seedset.

### 2.4 Statistical analysis

Differences between the cultivated and the wild *T. turgidum* accessions in crossability with the KU‐2103 tester and in their ability to cause genome doubling in the F_1_ hybrids were evaluated using a model selection approach based on generalized linear mixed model (GLMM) analysis. In this analysis, an alternative model that included a fixed effect was compared with a null model without a specified fixed effect, using the lme4 package (Bates et al., 2015) for R ver. 3.6 (R Core Team, 2019). Both models were generated using the *glmer* function of the package, which fits a fixed effect and random effects with a binomial error distribution on a logit link function based on a maximum likelihood estimation method. Model selection was performed based on the minimum Akaike Information Criterion (AIC) value, which is an estimate of the amount of information lost when a given model is used to approximate the process that generated the observed data. Overdispersion was estimated using the *overdisp_fun* function (Bolker, 2019). Departure of the alternative model from the null model was examined by a likelihood ratio test using the *anova* function. The theoretical marginal *R^2^* (the proportion of variance explained by the fixed effect) and conditional *R^2^* (the proportion of variance explained by the fixed effect and random effects) values of the alternative model were calculated using the *r.squarredGLMM* function of the MuMIn package (Bartoń, 2019; Nakagawa & Schielzeth, 2013). The R commander package (Fox, 2005) was used to conduct a chi‐squared test for the difference between cultivated and wild *T. turgidum* in terms of the proportions of the accessions that produced inviable F_1_ plants.

### 2.5 QTL analysis

QTL analysis was used to identify loci in the *T. turgidum* genome that influence the occurrence of genome doubling in F_1_ hybrids with *Ae. tauschii*. A *T. turgidum* accession that generated an F_1_ genotype with a high genome doubling frequency when crossed with the KU‐2103 tester (termed “high accession”) and an accession that generated an F_1_ genotype with a low genome doubling frequency when crossed with the KU‐2103 tester (termed “low accession”) were selected from the collection. To produce the segregant population, we first crossed the high accession (female) with the low accession (male). F_1_ plants of the cross between the high and low accessions were crossed with the KU‐2103 tester (male) to obtain seeds of trihaploid segregants (ABD genome) in which only the loci on the A and B genomes were segregated.

Seeds of the segregants (374 individuals), together with seeds of the high‐accession‐KU‐2103 F_1_ hybrid (one individual) and the low‐accession‐KU‐2103 F_1_ hybrid (one individual), were germinated in Petri dishes at 20°C, transplanted into individual pots in early winter, and then grown in an unheated greenhouse. The DNeasy plant Mini Kit (Qiagen) was used to extract total DNA from healthy leaves of individual plants. Each individual plant was subjected to DArTseq haplotyping at Diversity Arrays Technology Pty. Ltd. The hybrid genome doubling frequency in each segregant individual was measured as the selfed seedset rate. Selfing, seed and floret counting, and rate calculation were conducted as described above for measuring the hybrid genome doubling frequency.

DArTseq examines numerous genome fragments, aligns these fragments to a reference genome sequence, and produces a biallelic single nucleotide polymorphism (SNP) dataset. The SNP alleles were coded as “1” (present) or “0” (absent) and further screened for use based on the following criteria: (a) the SNPs must be physically mapped to the A or B genome of the *T. aestivum* reference genome sequence provided by Diversity Arrays Technology (wheat_ChineseSpring04); (b) the SNPs must be homozygotic in all individuals given their haploid nature (i.e., heterozygotic SNPs are considered erroneous); (c) the SNPs must have no missing values in the high‐accession‐KU‐2103 and low‐accession‐KU‐2103 F_1_ hybrids, because this information is required for determining the source *T. turgidum* accessions of the SNP alleles; and (d) the missing data percentage for each SNP must be less than 5%.

A linkage map was then constructed on the basis of the qualified SNPs and the MSTmap algorithm implemented in the ASMap package, using the *mstmap* function with the “bychr” and “anchor” options (Taylor & Butler, 2017; Wu et al., 2008). Genetic map distances were calculated based on the Kosambi function (Kosambi, 1944). The QTL analysis was carried out using the R/qtl package based on the model for recombinant inbred lines produced by selfing, because the haplotype data for the trihaploid segregants had two categories, with no possibility of heterozygosity (Broman et al., 2003). Conditional genotype probabilities were calculated using the *calc.genoprob* function (density, 1 cM), assuming a genotyping error rate of 0.001. The *sim.geno* function was used to impute missing genotypes, with 256 draws. Single‐QTL analyses were performed using the *scanone* function based on the EM algorithm (for the binary trait) and multiple imputation (for the quantitative trait). The statistical significance of putative QTLs was examined by 100,000‐fold permutation tests. Approximate 95% Bayesian credible intervals for the QTL locations were calculated using the *bayesint* function with the “expandtomarkers” option. QTL model fitting was done using the *makeqtl* and *fitqtl* functions. In multiple‐QTL modeling, the *addint* function was used to test pairwise interactions among QTLs.

### 2.6 Cytological observations

Meiotic cell divisions in the pollen mother cells (PMCs) were analyzed by the conventional aceto‐carmine squash method (Matsuoka & Nasuda, 2004) with some modifications. Immature anthers were collected from young spikes, fixed in a mixture of anhydrous ethanol and acetic acid (3:1), and stored at 4°C until use. For the tracking of cell division progression, anthers were sampled from consecutive primary or secondary florets arrayed along the rachis of a spike. For microscopy, fixed anthers were stained with aceto‐carmine at 20˚C for several hours and then squashed in a drop of 45% acetic acid. Cells were observed and imaged under a BX‐51 microscope equipped with a DP21 digital camera (Olympus, Tokyo, Japan).

## 3 RESULTS

### 3.1 Crossability

**FIGURE 1:**
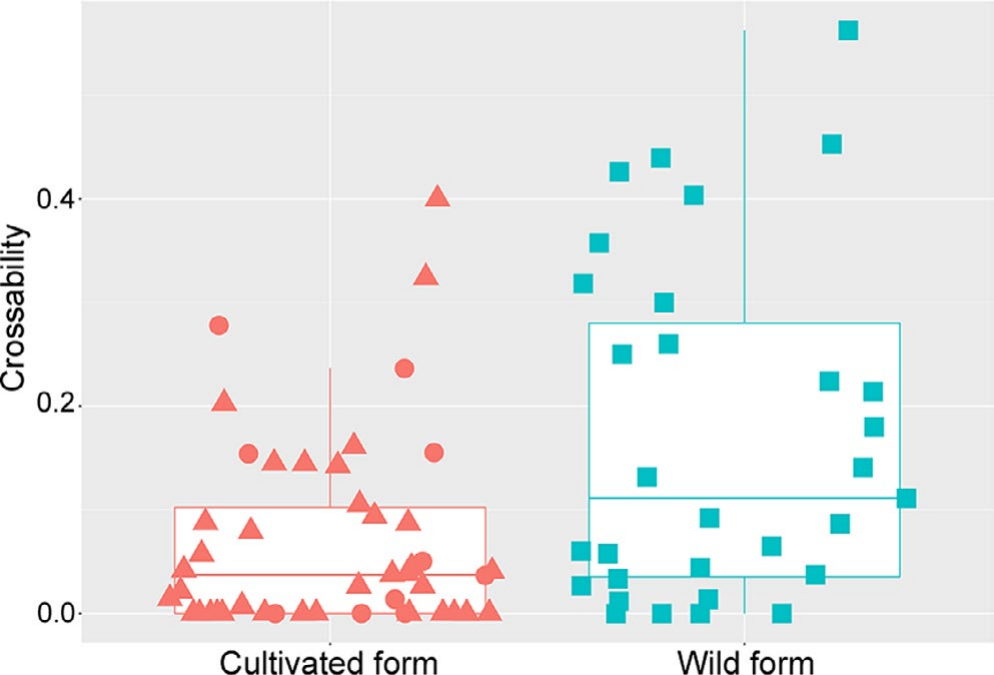
Box and dot plots of the crossability values of cultivated and wild forms of*Triticum turgidum*. In the cultivated form plot, triangles and circles denote free‐threshing and non‐free‐threshing accessions, respectively

The artificial cross‐experiment provided the crossability values for the 77 *T. turgidum* accessions (Figure 1; Table 1; Table S1). The overall median, mean, and standard deviation were 0.05, 0.11, and 0.14, respectively. In the cultivars (46 accessions), the crossability values varied from 0.00 to 0.40, and the median, mean, and standard deviation were 0.04, 0.07, and 0.09, respectively. In the hulled cultivars (*T. turgidum* subsp. *dicoccum*), the crossability values ranged from 0.00 to 0.28. Sixteen of the 46 cultivars (37.0%) had a crossability value of 0.00. In the 31 wild accessions, the crossability values varied from 0.00 to 0.56, and the median, mean, and standard deviation were 0.11, 0.17, and 0.16, respectively. Four of the 31 wild accessions (12.9%) had a crossability value of 0.00. In general, crossability with the *Ae. tauschii* tester tended to be higher in the wild accessions than in the cultivars, but the value ranges overlapped widely between the two groups.

**TABLE 1:** *Triticum turgidum* crossability values obtained in the artificial cross‐experiment

Category	No. of accessions	Median	Mean	Standard deviation	Range
Overall	77	0.05	0.11	0.14	0.00–0.56
Cultivars (all)	46	0.04	0.07	0.09	0.00–0.40
Hulled cultivars	10	0.04	0.09	0.11	0.00–0.28
Free‐threshing cultivars	36	0.03	0.06	0.09	0.00–0.40
Wild accessions	31	0.11	0.17	0.16	0.00–0.56

To further examine the difference in crossability between the cultivars and wild accessions, we analyzed the seedset data by GLMM analysis (Table S2). The GLMM used “form” (i.e., cultivar vs. wild) as the fixed effect and “accession” as the random effect. Comparison of the alternative and null models revealed that inclusion of the fixed effect improved the goodness of prediction in the alternative model (AIC = 766.7) relative to that of the null model (AIC = 773.9) (Table 2). The departure of the alternative model from the null model was significant, indicating that “form” had a significant effect on the prediction of the numbers of seeds set in the spikes (likelihood ratio test, *p* = 0.002). Based on the results of the *overdisp_fun* function (Bolker, 2019), none of the models were overdispersed: the ratios between the residual deviance and the residual degrees of freedom were close to 1.00 (0.83 for the alternative model and 0.82 for the null model), and the *p* values were >0.05 (0.95 for the alternative model and 0.96 for the null model). The marginal and conditional *R^2^* values of the alternative model were 0.06 and 0.49, respectively.

**TABLE 2:** Linear mixed models to evaluate differences in the crossability with the KU‐2103 tester between the cultivated and wild forms of *Triticum turgidum* ^a^

		Model	
		Null	Alternative
Number of observed spikes		177	177
Number of accessions		77	77
Coefficient of fixed effect	Intercept	−3.06	−3.58
	Wild form		1.32
*z* value for fixed effect	Intercept	−13.02	−12.2
	Wild form		3.09
Variance for random effect	Accession	3.25	2.80
AIC		773.9	766.7
Likelihood ratio test (null model vs. alternative model)	*χ* ^2^		9.24
	Degree of freedom		1
	*p*		0.00
Marginal *R* ^2^			0.06
Conditional *R* ^2^			0.49

^a^ Blank denotes not applicable.

### 3.2 Hybrid viability

Thirty‐three *T. turgidum* accessions produced at least one viable F_1_ plant in the artificial crosses (Table S1), while 44 accessions produced no viable F_1_ plants. Of these, 20 accessions did not set seeds, and 24 accessions set seeds but produced F_1_ plants that were not viable due to hybrid abnormalities, such as germination failure or severe dwarfness (Table S1). The proportion of the accessions that set seeds in the artificial crosses but produced abnormal F_1_ plants was higher in the wild accessions (15 out of 27 accessions, 55.6%) than in cultivars (nine out of 30 accessions, 30.0%); however, this difference was not statistically significant (chi‐squared test, *p* = 0.05).

### 3.3 Hybrid genome doubling frequency

**FIGURE 2:**
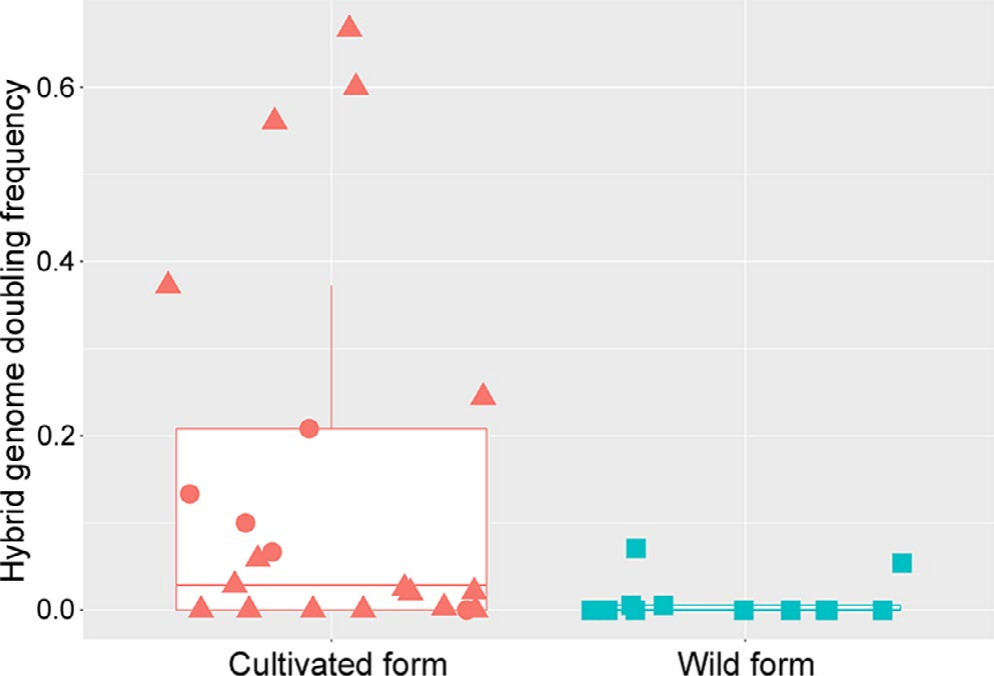
Box and dot plots of the hybrid genome doubling frequencies of cultivated and wild forms of*Triticum turgidum*. In the cultivated form plot, triangles and circles denote free‐threshing and non‐free‐threshing accessions, respectively

In cultivar‐derived F_1_ hybrids (21 genotypes), the genome doubling frequency varied from 0.00 to 0.67, and the median, mean, and standard deviation were 0.03, 0.15, and 0.22, respectively (Figure 2; Table 3; Table S1). Notably, three free‐threshing accessions produced F_1_ genotypes that had particularly high hybrid genome doubling frequencies (>0.5): *T. turgidum* subsp. *carthlicum* (KU‐138) and *T. turgidum* subsp. *durum* (PI73306 and LDN). The frequency of the LDN–KU‐2103 F_1_ (0.56) was consistent with the previously reported value of 0.53 (Matsuoka et al., 2013). F_1_ genotypes derived from the hulled cultivar accessions had hybrid genome doubling frequencies of <0.21. In wild‐accession‐derived F_1_ hybrids (12 genotypes), the genome doubling frequency varied from 0.00 to 0.07, and the median, mean, and standard deviation were 0.00, 0.01, and 0.02, respectively (Figure 2; Table 3; Table S1). Only four wild accessions produced F_1_ genotypes that set seeds: KU‐1921, KU‐1976B, KU‐14417, and MORI116. Clearly, the hybrid genome doubling frequency tended to be higher in the cultivar‐derived F_1_ genotypes than in the wild‐accession‐derived F_1_ genotypes.

**TABLE 3:** Hybrid genome doubling frequencies in the *Triticum turgidum*–KU‐2103 F_1_ genotypes

Category	No. of genotypes	Median	Mean	Standard deviation	Range
Overall	33	0.01	0.10	0.18	0.00–0.67
Cultivar‐derived (all)	21	0.03	0.15	0.22	0.00–0.67
Hulled cultivar‐derived	5	0.10	0.10	0.08	0.00–0.21
Free‐threshing‐cultivar‐derived	16	0.02	0.16	0.24	0.00–0.67
Wild‐accession‐derived	12	0.00	0.01	0.02	0.00–0.07

The difference in the hybrid genome doubling frequency between the cultivar‐ and the wild‐accession‐derived F_1_ genotypes was further examined by GLMM analysis of selfed seedset data (Table S3). In the GLMM, “form” (i.e., cultivar‐ vs. wild‐accession‐derived) was the fixed effect, whereas “parental *T. turgidum* accession” and “individual F_1_ plant ID” were the random effects. Goodness of prediction was improved in the alternative model (AIC = 1,367.0) relative to that of the null model (AIC = 1,373.2) (Table 4). The departure of the alternative model from the null model was significant, indicating that “form” had a significant effect on the prediction of the numbers of seeds set in the spikes (likelihood ratio test, *p* = 0.004). Based on the results of the *overdisp_fun* function (Bolker, 2019), none of the models were overdispersed; the ratios between the residual deviance and the residual degrees of freedom were close to 1.00 (1.05 for the alternative model and 1.03 for the null model), and the *p* values were >0.05 (0.19 for the alternative model and 0.25 for the null model). The marginal and conditional *R^2^* values of the alternative model were 0.19 and 0.78, respectively.

**TABLE 4:** Linear mixed models to evaluate differences in the hybrid genome doubling frequencies between the cultivar‐derived and wild‐accession‐derived F_1_ genotypes^a^

		Model	
		Null	Alternative
Number of observed spikes		531	531
Number of maternal *T. turgidum* accessions		33	33
Number of plants		88	88
Coefficient of fixed effect	Intercept	–5.03	–3.70
	Wild form		–3.65
*z* value for fixed effect	Intercept	–6.83	–5.24
	Wild form		–2.85
Variance for random effect	Parental *T. turgidum* accession	10.92	8.53
	Individual F_1_ plant ID	0.25	0.25
AIC		1,373.2	1,367.0
Likelihood ratio test (null model vs. alternative model)	*χ* ^2^		8.18
	Degree of freedom		1
	*p*		0.00
Marginal *R* ^2^			0.19
Conditional *R* ^2^			0.78

^a^ Blank denotes not applicable.

### 3.4 QTLs for hybrid genome doubling

**FIGURE 3:**
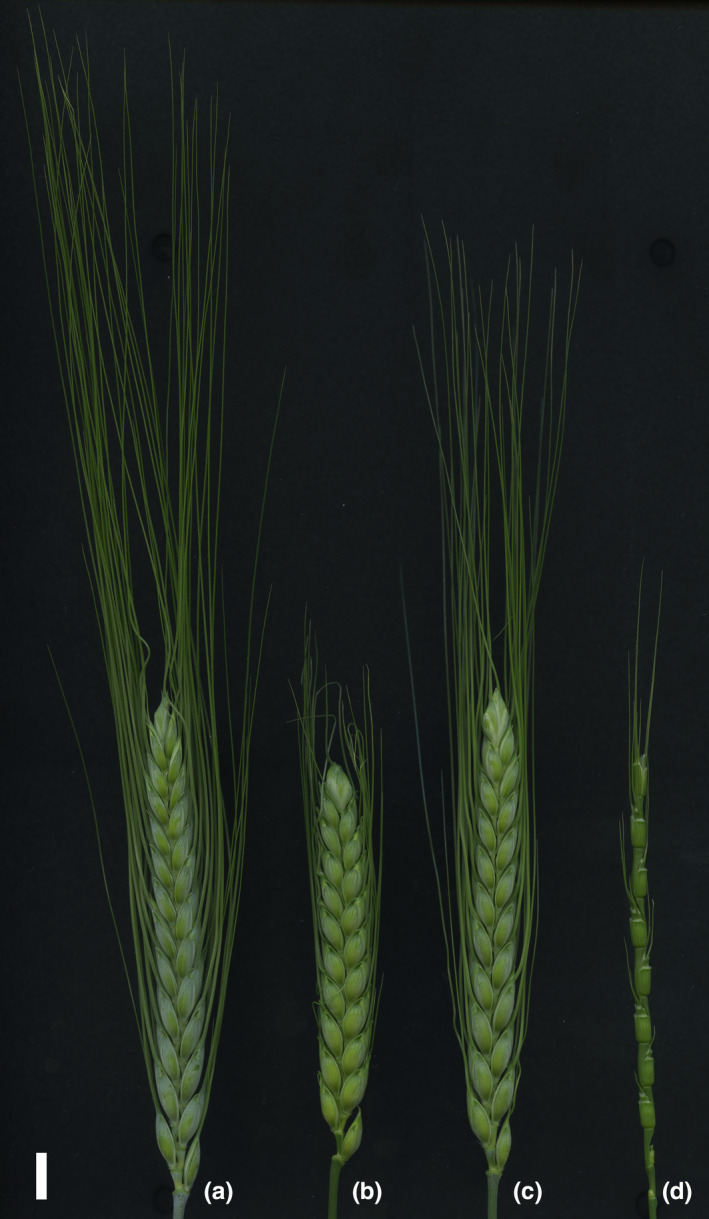
Spikes of the accessions used in the QTL analysis. (a) LDN, (b) KU‐9882, (c) F_1_hybrid between LDN and KU‐9882, (d) KU‐2103.*Bar* = 1 cm

The hybrid genome doubling frequency spectrum suggested that some *T. turgidum* cultivars might have specific alleles of the genes that regulate the trait. To examine the genetic basis of hybrid genome doubling, we performed a QTL analysis on a population of trihaploid segregants (ABD genome) in which only the loci on the A and B genomes had segregated. LDN was selected as the high accession (the hybrid genome doubling frequency of the LDN‐derived F_1_ = 0.56), whereas KU‐9882 was selected as the low accession (the hybrid genome doubling frequency of the KU‐9882‐derived F_1_ = 0.00). These accessions had similar crossability values in the artificial cross‐experiment (0.40 for LDN and 0.32 for KU‐9882) (Figure 3).

**FIGURE 4:**
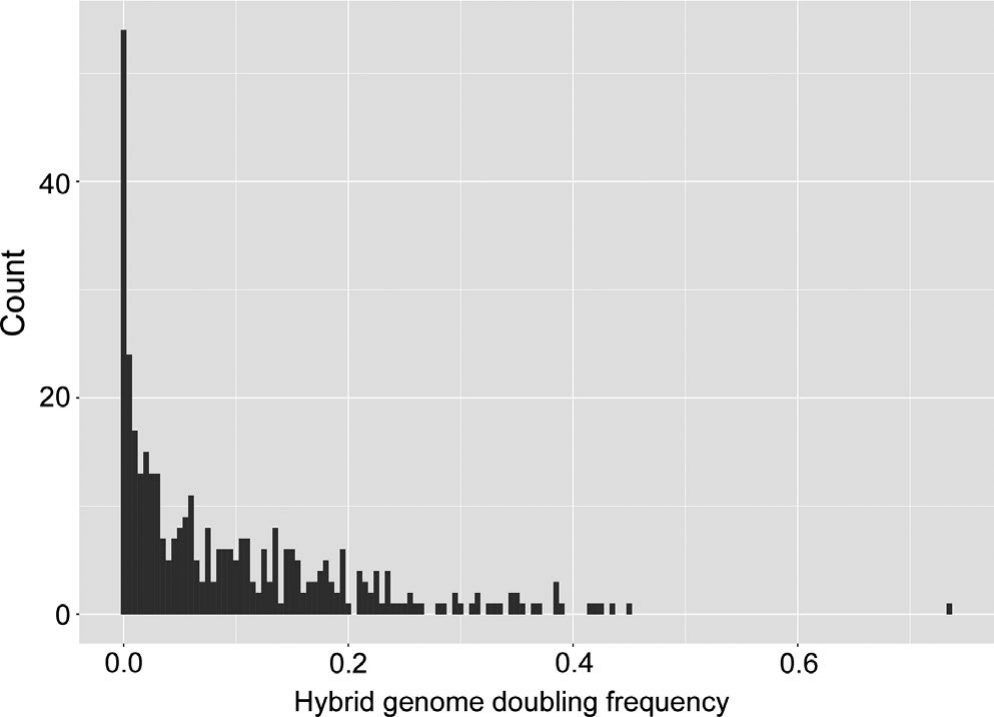
Distribution of the hybrid genome doubling frequencies in the 374 trihaploid segregants obtained by crossing LDN–KU‐9882 F_1_plants with the KU‐2103 tester. Bin width was set to 0.005

In total, 374 segregants were obtained. The hybrid genome doubling frequency of the segregants varied from 0.00 to 0.73, and the median, mean, and standard deviation were 0.06, 0.10, and 0.11, respectively. The frequency distribution was left‐skewed, with 54 segregants having a frequency of 0.00 (Figure 4). DArTseq haplotyping and subsequent polymorphism screening yielded 1,110 qualified SNPs for linkage map construction. The MSTmap algorithm generated 50 linkage groups, and after removing those that had six or fewer SNPs, a reasonable genetic map of the 14 chromosomes was obtained. The map had 1,061 SNPs in total, and the number of SNPs that anchored to each chromosome was 70 (1A), 76 (2A), 73 (3A), 72 (4A), 70 (5A), 45 (6A), 91 (7A), 77 (1B), 111 (2B), 77 (3B), 57 (4B), 88 (5B), 79 (6B), and 75 (7B). The total map length was 1679.8 cM, and the average spacing and maximum spacing were 1.6 and 23.5 cM, respectively (Figure S1).

**FIGURE 5:**
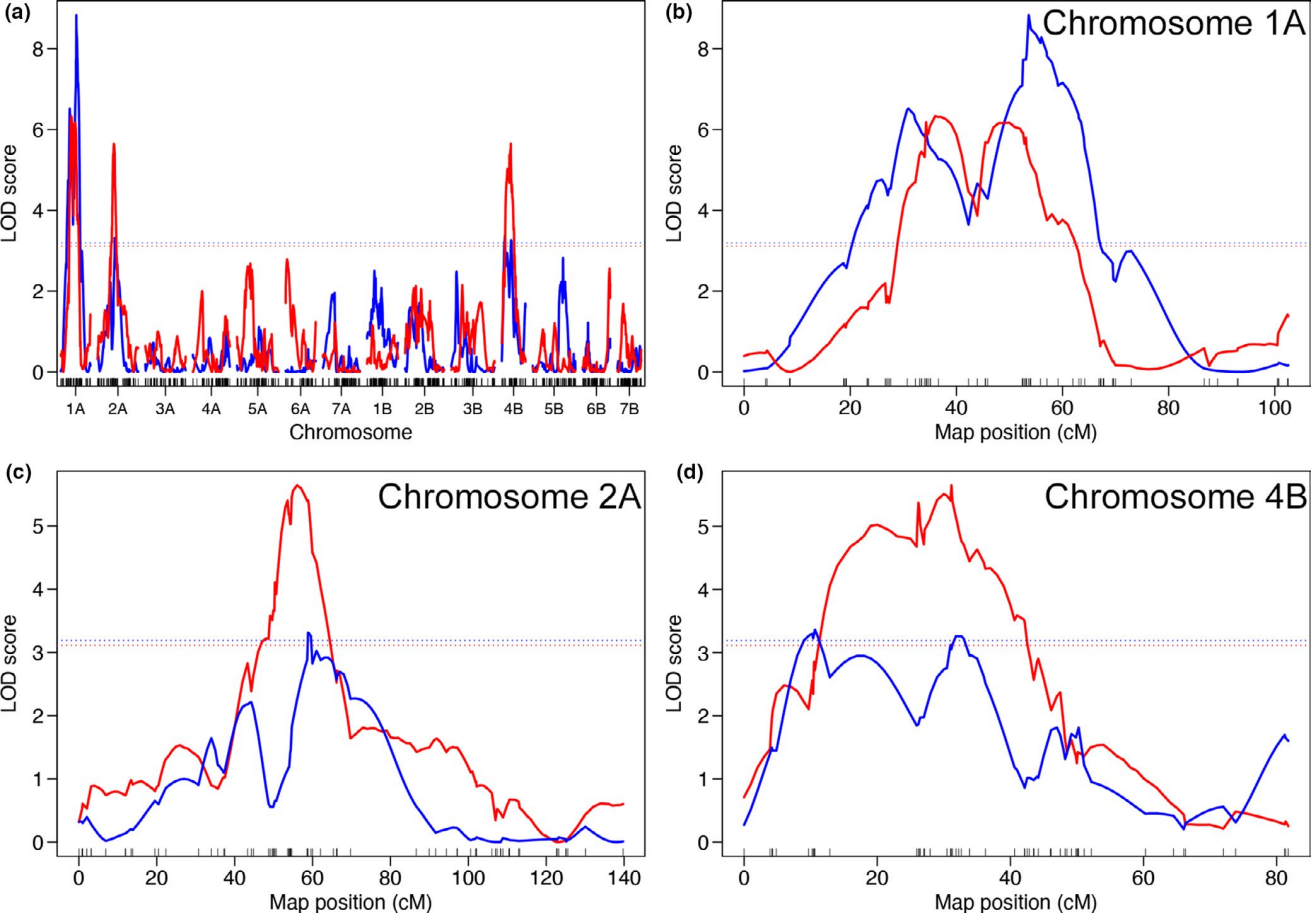
QTL analysis of hybrid genome doubling using the trihaploid segregant population. Blue and red colors represent the results for the binary trait and quantitative traits, respectively. Horizontal dashed lines indicate significant LOD scores determined by permutation. (a) All chromosomes. (b) chromosome 1A. (c) Chromosome 2A. (d) Chromosome 4B

The hybrid genome doubling frequency distribution had a spike at 0.00 (Figure 4). Therefore, we first transformed the original trait into a binary trait (defined as “1” when the original phenotype was >0.00 and “0” when the original phenotype was 0.00) and a quantitative trait (the same as the original phenotypes, except that 0.00 was treated as missing), and then separately performed QTL analyses for each binary and quantitative trait (Broman, 2003) (Table S4). Single‐QTL analysis of the binary trait revealed three significant QTLs located on chromosomes 1A, 2A, and 4B (Figure 5; Table 5). Analysis of the quantitative trait also revealed three significant QTLs in the similar regions of the same chromosomes. Multiple‐QTL modeling showed that additive QTL models based on the three significant QTLs explained 14.9% (for the binary trait) and 16.6% (for the quantitative trait) of the phenotype variances (Table 6). In the case of the binary trait, the locus on chromosome 1A had a particularly strong effect [logarithm of odds (LOD) = 8.2; proportion of variance explained (PVE) = 9.0%; *p* = 8.16e−10, drop‐one‐QTL‐at‐a‐time analysis], whereas the effects of the other two QTLs were small. In the three‐QTL model for the quantitative trait, the locus on chromosome 1A also had a strong effect (LOD = 6.4; PVE = 8.1%; *p* = 6.00e−10, drop‐one‐QTL‐at‐a‐time analysis), whereas the other two QTLs had very small effects. In both traits, the estimated effects of the three QTLs were negative, indicating that the alleles of the high accession LDN had a positive influence on hybrid genome doubling. Generally, between‐loci interactions had small effects in the model for the binary trait, whereas in the model for the quantitative trait, the interaction of the 1A locus with each of the other two loci had a moderate effect (LOD = 2.2 and PVE = 2.6% for the 1A–2A loci interaction and LOD = 1.4 and PVE = 1.7% for the 1A–4B loci interaction) (Table 6).

**TABLE 5:** QTLs found by single‐QTL analysis of the binary and quantitative traits of hybrid genome doubling

Trait	QTL name	Chromosome	Position (cM)	LOD score	*p*	%var	Approximate 95% Bayesian credible interval (cM) and DArTseq marker name
Binary	b.1A	1A	53.6	8.83	0.00	10.3	52.4; 1096317|F|0–25:T > C_1A
							62.0; 4991333|F|0–18:T > G_1A
	b.2A	2A	58.8	3.32	0.04	4.0	37.5 3222362|F|0–13:A > G_2A
							86.6 1080633|F|0–32:C > A_2A
	b.4B	4B	10.6	3.36	0.03	4.1	4.8 3064433|F|0–16:C > A_4B
							40.6 3028936|F|0–26:C > T_4B
Quantitative	q.1A	1A	36.0	6.33	0.00	8.7	33.1 5325244|F|0–25:T > G_1A
							53.2 1228810|F|0–10:T > A_1A
	q.2A	2A	56.0	5.64	0.00	7.8	50.7 1720560|F|0–14:C > G_2A
							59.6 1381499|F|0–18:C > T_2A
	q.4B	4B	31.1	5.65	0.00	7.8	12.9, 3955316|F|0–19:A > C_4B
							36.2, 3222467|F|0–7:C > T_4B

Note

*p* denotes the genome‐scan‐adjusted *p*‐value for the LOD peak.

%var denotes the estimated proportion of the phenotype variance explained by the QTL.

**TABLE 6:** Multiple‐QTL models for the binary and quantitative traits of hybrid genome doubling

Model Trait	QTL/QTL combination name	Chromosome	Position (cM)	LOD score	%var by QTL	*P*	Estimated effect (standard error)
Binary	b.1A	1A	53.6	8.2	9.0	8.16e−10	−1.05 (0.20)
	b.2A	2A	58.8	1.9	2.0	0.00	−0.53 (0.18)
	b.4B	4B	10.6	1.0	1.1	0.03	−0.39 (0.18)
Quantitative	q.1A	1A	36.0	6.4	8.1	6.00e−8	−0.03 (0.01)
	q.2A	2A	56.0	0.8	1.0	0.06	−0.02 (0.01)
	q.4B	4B	31.1	0.3	0.4	0.24	−0.01 (0.01)
	q.1A:q.2A	na	na	2.2	2.6	0.00	na
	q.1A:q.4B	na	na	1.4	1.7	0.01	na
	q.2A:q.4B	na	na	0.1	0.2	0.44	na

Note

*p* denotes the drop‐one‐QTL‐at‐a‐time analysis of variance *p*‐value for the LOD peak.

%var denotes the estimated proportion of the phenotype variance explained.

na denotes not applicable for this category.

In the binary trait model, LOD score (relative to the no QTL model), %var by all terms in the model, and *p*‐value based on the LOD score were 13.1, 14.9, and 5.1e−13, respectively.

In the quantitative trait model, LOD score (relative to the no QTL model), %var by all terms in the model, and *p*‐value based on the LOD score were 12.6, 16.6, and 1.4e−12, respectively.

### 3.5 Male sporogenesis in the KU‐9882–KU‐2103 F_1_ hybrids

**FIGURE 6:**
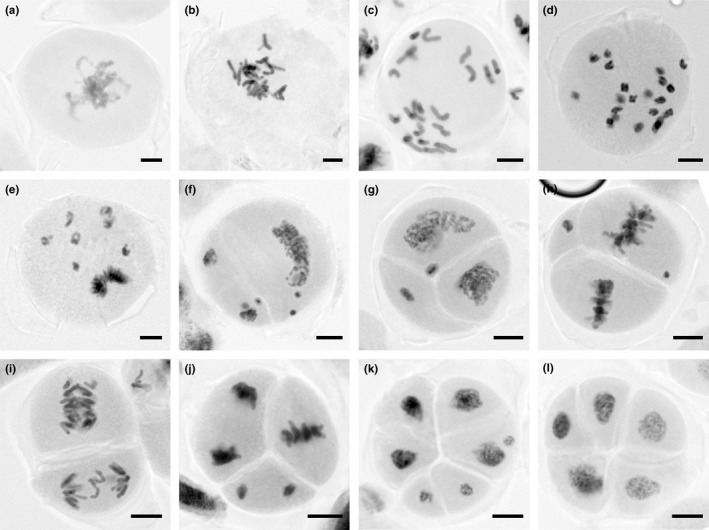
Aberrant nonreductional meiosis in the PMCs of KU‐9882–KU‐2103 F_1_plants. (a) Prophase. No univalents are visible. (b) Prophase. Univalents are visible. (c) Prophase. Univalents are observed in three clusters. (d) Univalents split into sister chromatids. (e) Univalents move to the multiple spindle poles. (f) Restitutive nuclei. Cytokinesis is taking place. (g) Restitutive nuclei in multiple daughter cells. (h) Chromosomes are condensed and aligned at the spindle equator in the daughter cells. (i) Anaphase. (j) Heterochronic daughter cells at telophase and metaphase. (k) Cytokinesis is taking place in the daughter cells. (l) Pentad.*Bar* = 20 µm

Triploid F_1_ hybrids between LDN and KU‐2103 set seeds because they produced unreduced gametes at a high rate through nonreductional meiosis. During male gamete formation, PMCs undergo a single cell division and produce pollen dyads instead of tetrads (Matsuoka et al., 2013). However, why triploid F_1_ hybrids between the low‐accession KU‐9882 and the KU‐2103 tester produced no seeds was not clear. To address this question, we microscopically analyzed PMC division in the KU‐9882–KU‐2103 F_1_ hybrids. Anthers sampled from three series of consecutive florets that covered the prophase to the daughter cell stage were used. Observation of a few hundred PMCs revealed that the F_1_ hybrids underwent aberrant nonreductional meiotic divisions and predominantly produced pollen polyads (Figure 6). At prophase, 21 univalent chromosomes were visible (Figure 6a,b). In several cells, the univalents were observed in a few clusters (Figure 6c). No PMCs displayed univalents that were aligned to the spindle equator. Subsequently, the univalents split into sister chromatids, moved to the multiple spindle poles, and formed restitution nuclei (Figure 6d,e). After this stage, the cells underwent irregular cytokinesis, chromosome condensation, and aberrant divisions (Figure 6f–k). Finally, atypical polyad daughter cells were observed (Figure 6l).

## 4 DISCUSSION

### 4.1 Possible key role of hybrid genome doubling in the evolution of common wheat

In the widely accepted scenario for the origin of common wheat, the cultivated form, not the wild form of *T. turgidum,* is the female progenitor (Kihara, 1966; Kihara, 1975). This hypothesis is based mainly on the fact that wild *T. aestivum* has never been found, indicating that, under natural conditions, successful hybridization between wild *T. turgidum* and *Ae. tauschii* occurs rarely. Cultivated *T. turgidum* and *Ae. tauschii* are known to co‐exist in the wheat fields of northern Iran but natural hybridization between these species has never been observed (Matsuoka, et al., 2007). In a previous series of artificial cross‐experiments, wild *T. turgidum* was often involved in the expression of reproductive barrier phenotypes (such as chlorosis and necrosis) in F_1_ hybrids with *Ae. tauschii*. This suggested that strong reproductive isolation through postzygotic mechanisms might explain why wild *T. aestivum* does not exist (Kihara, 1966; Kihara, 1975). However, the genetic relationships between the *Ae. tauschii* testers and the D genome of common wheat were not addressed in those experiments. Thus, the interpretation of the results in terms of understanding the origin of common wheat is not straightforward.

In the present study, we used the KU‐2103 tester in the artificial cross‐experiment and found that (a) the crossability values tended to be lower in cultivars (0.00–0.40) than in wild accessions (0.00–0.56), although the ranges widely overlapped; (b) the proportion of the accessions that set seeds in the artificial cross, but provided inviable F_1_ plants due to reproductive barrier phenotypes was similar between cultivars and wild accessions; and (c) the hybrid genome doubling frequency greatly varied in cultivar‐derived F_1_ genotypes (0.00–0.67), whereas it was generally low in wild‐form‐derived F_1_ genotypes (≤0.07). KU‐2103 was a suitable tester to mimic the genetic conditions under which early‐stage common wheat evolution proceeded because of its short genetic distance to the common wheat D genome (Wang et al., 2013). For this reason, the findings of the present study may provide novel insights into the evolution of common wheat.

Cultivated and wild *T. turgidum* clearly differed in the ability to cause hybrid genome doubling in the F_1_ with KU‐2103:14 out of 20 cultivars (70.0%) produced F_1_ genotypes that set seeds, including genotypes with a particularly high seedset rate (>0.5), whereas only four out of 12 wild accessions (33.3%) yielded F_1_ genotypes that set seeds and only at low rates (≤0.07). In contrast, the differences in crossability and the proportion of the accessions that produced F_1_ genotypes having reproductive barrier phenotypes were less prominent. Therefore, the probability of allohexaploid formation through hybridization with the ancestral *Ae. tauschii* might have been higher for the cultivated form of *T. turgidum* than for the wild form, while other reproductive conditions were likely similar. Thus, the present study provides empirical support for the hypothesis that cultivated *T. turgidum* is the female progenitor of common wheat. Importantly, our findings underscore a possible key role of hybrid genome doubling in the evolution of common wheat, as the enhanced hybrid genome doubling ability in cultivated *T. turgidum* must have positively influenced the likelihood of successful formation of allohexaploid offspring.

An obvious caveat here is that our artificial cross‐experiment relied on a single *Ae. tauschii* tester. In general, the genotypes of the *Ae. tauschii* accessions influence the crossability with *T. turgidum* and the viability and genome doubling frequency of the F_1_ hybrids (Matsuoka & Takumi, 2017; Matsuoka, et al., 2007). Thus, the phenotypes of these traits may vary in the F_1_ hybrids, even among the *Ae. tauschii* testers that are genetically close to the common wheat D genome. For this reason, further studies that use testers other than KU‐2103 are required to evaluate the validity of our inference regarding the importance of hybrid genome doubling in the evolution of common wheat. Additionally, in this regard, we note that results similar to our findings were obtained in the previous studies. For example, Nishikawa (1964) performed an artificial cross‐experiment using 11 *T. turgidum* accessions (one wild and ten cultivated accessions) and five *Ae. tauschii* accessions (including one artificial autotetraploid accession). Consistent with our findings, the reported mean crossability value (over the five *Ae. tauschii* testers) was larger for the wild *T. turgidum* accession (0.334) than for the cultivated accessions (0.073–0.165). In addition, F_1_ genotypes derived from three out of the five wild forms grew normally, but did not set F_2_ seeds. Thus, a relatively high crossability with *Ae. tauschii* and a reduced ability to cause genome doubling in F_1_ offspring might be common phenotypes in wild *T. turgidum*.

### 4.2 Genetic basis of hybrid genome doubling

Hybrid genome doubling through unreduced gamete fusion is an important mechanism in plant allopolyploid evolution (Ramsey & Schemske, 1998). Therefore, clarifying the mechanical underpinning of genome doubling in the *T. turgidum*‐*Ae. tauschii* F_1_ hybrids is essential to understand the evolution of common wheat. We addressed this issue by conducting QTL analysis. The three QTLs found for each binary and quantitative trait were mapped to similar locations on the 1A, 2A, and 4B chromosomes, suggesting that the same loci are responsible for these traits. These QTLs are novel because one QTL for hybrid genome doubling found in a previous study was situated on chromosome 3B (Hao et al., 2014). In the present study, we did not identify the QTL on chromosome 3B which may be because we used a low accession different from the ones of Hao et al. (2014), although the high accession, LDN, was the same. Another possible explanation for the absence of the QTL on chromosome 3B in our study may be that this QTL is not involved in hybrid genome doubling when the KU‐2103 tester, instead of the *Ae. tauschii* tester used by Hao et al. (2014), is used as the paternal parent. If this were the case, the genetic mechanism that underlies hybrid genome doubling would involve interactions between the maternal and paternal loci in the F_1_ genotypes. The extent to which such possible interactions are important for hybrid genome doubling remains to be addressed. Importantly, unlike the low accessions used in the previous study, KU‐9882 produced completely sterile F_1_ hybrids with the *Ae. tauschii* tester. Therefore, the alleles of KU‐9882 at those QTLs might have a strong negative influence on hybrid genome doubling.

Cytological observations revealed that KU‐9882–KU‐2103 F_1_ plants were male sterile because they were not capable of producing functional unreduced gametes due to aberrations in nonreductional meiosis. Previously, we observed PMCs of LDN–KU‐2103 F_1_ plants and found that they produced functional unreduced gametes at a high frequency through first division restitution (i.e., skipping of the first division of normal meiosis; Matsuoka et al., 2013). Interestingly, the nonreductional meiosis in KU‐9882–KU‐2103 F_1_ clearly differed from that in LDN–KU‐2103 F_1_ in that the 21 univalents did not align at the spindle equator prior to the restitution phase (Figure 6c). This suggests that the bipolar spindle structure might not be formed on the univalents of KU‐9882–KU‐2103 F_1_ plants. If this were the case, the QTLs found in the present study might be involved in the formation of the bipolar spindle on the univalents. To date, the meiotic genes that have been functionally characterized in wheat include *RecQ‐7*, which is located on chromosome 2A (Gardiner et al., 2019). However, this helicase gene is involved in gene conversion in normal meiosis and thus may not be eligible as a candidate gene for the chromosome 2A QTL.

In conclusion, this study suggests that the enhanced ability of cultivated *T. turgidum* to cause genome doubling in F_1_ hybrids with *Ae. tauschii* might have increased the probability of the emergence of common wheat. It also suggests that this enhanced ability might be the result of alterations in a relatively small number of loci. The nature of such alterations remains obscure, but they may have been mutations at the QTLs after the domestication of *T. turgidum*. Alternatively, the alterations might have occurred through the introgression of alleles that already existed in some unfound wild *T. turgidum* populations. Further studies on those QTLs may increase our understanding of the evolution of common wheat.

## CONFLICT OF INTEREST

No conflict of interest has been declared by the authors.

## AUTHOR CONTRIBUTIONS

**Yoshihiro Matsuoka:** Conceptualization (lead); data curation (lead); formal analysis (lead); funding acquisition (lead); investigation (lead); methodology (lead); project administration (lead); resources (equal); supervision (lead); validation (lead); visualization (lead); writing – original draft (lead); writing – review and editing (lead). **Naoki Mori:** Conceptualization (supporting); resources (equal); writing – original draft (supporting); writing – review and editing (supporting).

## Supporting information

Figure S1

Table S1

Table S2

Table S3

Table S4

## Data Availability

The data that supports the findings of this study are available in the supporting information of this article.
