# Supplementary figures and images for: Reproductive and genetic roles of the maternal progenitor in the origin of common wheat (Triticum aestivum L.)
Source: Ecol Evol. 2020 Dec 2;10(24):13926–37. doi: 10.1002/ece3.6985 (PMC7771132; doi:10.1002/ece3.6985)

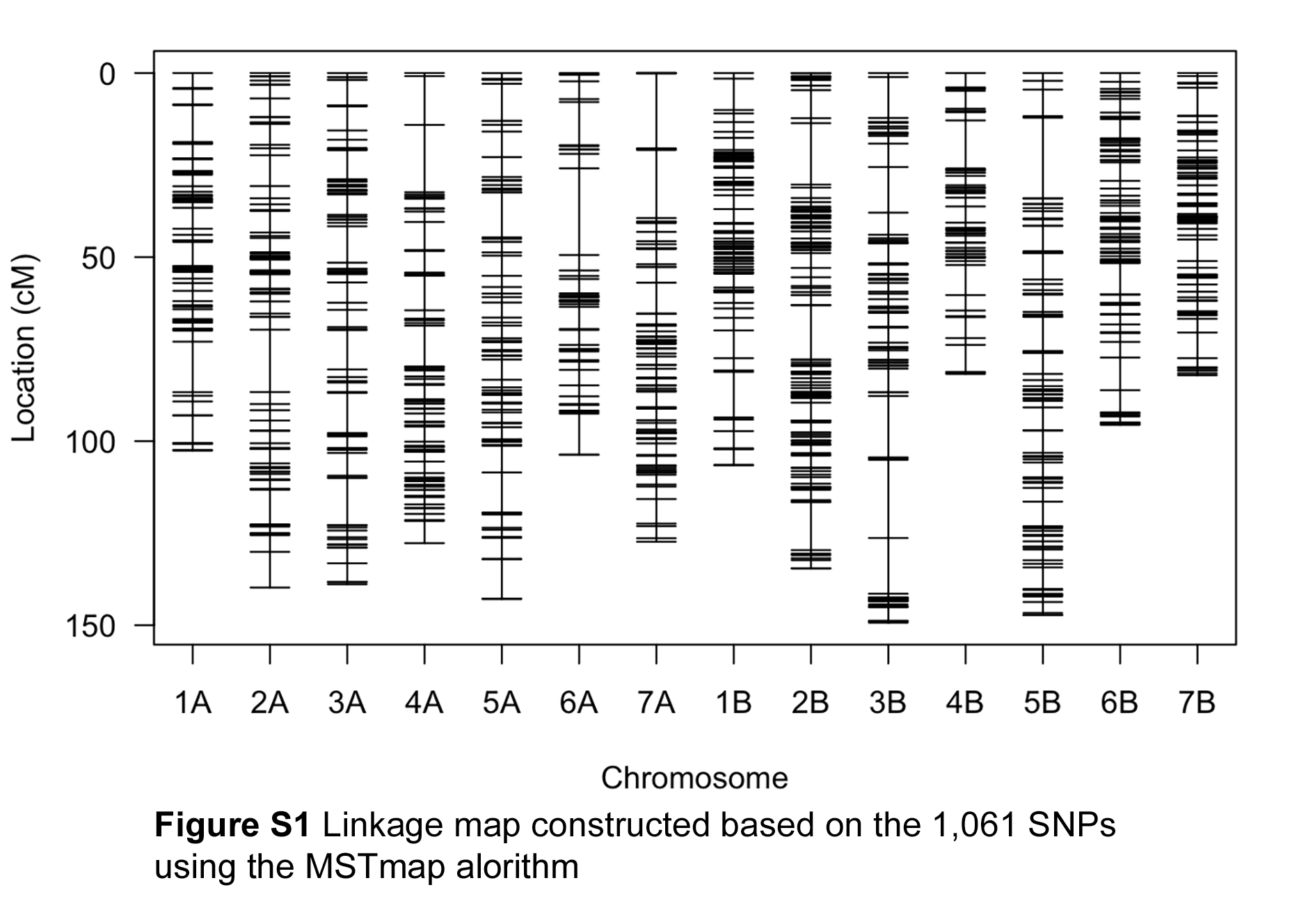

Supplement: Supplementary file 1 — Figure S1 [file ECE3-10-13926-s001.tif]
